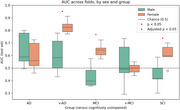# Investigating sex differences in connected speech across the Alzheimer's disease spectrum using machine learning

**DOI:** 10.1002/alz70857_101206

**Published:** 2025-12-24

**Authors:** Natasha Clarke, Christophe Bedetti, Pierre‐Briac Metayer, Simona Maria Brambati

**Affiliations:** ^1^ Université de Montréal, Montréal, QC, Canada; ^2^ Centre de Recherche de l'Institut Universitaire de Gériatrie de Montréal, Montréal, QC, Canada

## Abstract

**Background:**

Alterations in connected speech (CS), such as when describing a picture, have been identified in Alzheimer's disease (AD) and could act as markers of subjective (SCI) and mild cognitive impairment (MCI), and offer opportunities for therapeutic intervention. Machine learning has shown promise in classifying individuals along the AD spectrum using CS features. However, subtle sex differences in language may influence symptom presentation and classification accuracy. We investigated the impact of sex on CS and classification performance across the AD spectrum.

**Methods:**

We analysed Cookie Theft scene descriptions from 751 participants in the CCNA COMPASS‐ND cohort. Forty lexical, semantic and syntactic CS features were extracted using a Python‐based pipeline, and used to train ten logistic regression models. Classification of AD, vascular‐AD (v‐AD), MCI, vascular‐MCI (v‐MCI), and SCI versus cognitively unimpaired (CU) participants was performed separately for men and women using 5‐fold cross‐validation. Age and education were regressed from features within each fold. Mean area under the curve (AUC) was calculated and sex differences in classification performance assessed using Bonferroni‐corrected *t*‐tests. Features were then ranked based on standardised model coefficients.

**Results:**

Women were classified with higher AUC than men in SCI, MCI, and v‐AD, though only MCI remained significant after correction (*p* = 0.02). SCI and MCI classifications performed above chance for women, but below chance for men (Figure 1). We therefore focused on important features for v‐AD classifications, which performed above chance for both sexes, using feature rankings. Compared to CU men, men with v‐AD produced fluent speech that lacked detail, with more words indicating lexical access difficulties (e.g “remember”), yet syntactically complex speech (more subordinate phrases and left branching children), which may indicate compensation for lexical difficulties. Compared to CU women, women with v‐AD produced non‐fluent speech with more filled pauses (e.g. “um”), that was repetitive and relied on more common words and phrases, yet also syntactically complex (more subordinate phrases and coordinating conjunctions).

**Conclusions:**

Sex‐stratified classification models revealed differences in performance, with implications for research and clinical applications. Linguistic markers may be more sensitive for women along the AD spectrum, highlighting the importance of sex‐stratified analyses.